# Exploring knowledge, attitude, practices and barriers toward colorectal cancer screening in the United Arab Emirates: a mixed-methods study

**DOI:** 10.3389/fpubh.2025.1548258

**Published:** 2025-09-17

**Authors:** Iffat Elbarazi, Zufishan Alam, Mouza Alshebli, Lamia Alsunaidi, Ghada S. M. Al-Bluwi, Fayeza Faheem, Aminu S. Abdullahi, Luai A. Ahmed, Fatma Al-Maskari

**Affiliations:** ^1^College of Medicine and Health Sciences, Institute of Public Health, United Arab Emirates University, Al-Ain, United Arab Emirates; ^2^School of Health Sciences, Hamdan Bin Mohammed Smart University, Dubai, United Arab Emirates; ^3^Department of Internal Medicine, College of Medicine and Health Sciences, United Arab Emirates University, Al-Ain, United Arab Emirates; ^4^Zayed Centre for Health Sciences, United Arab Emirates University, Al Ain, United Arab Emirates

**Keywords:** Arab, health behavior (MeSH), colorectal cancer, colonoscopy, mixed method

## Abstract

**Background:**

Colorectal cancer (CRC) is a major contributor to cancer-related morbidity and mortality worldwide. In the United Arab Emirates (UAE), the CRC screening program was introduced in 2014, and it was expected that the program would face challenges, including low participation and poor adherence. However, there is limited research to document awareness and uptake of colorectal cancer screening among the UAE population.

**Aim:**

This study aimed to assess the knowledge and behaviors of the adult Emirati population in Al-Ain, UAE, toward CRC screening, along with the barriers affecting uptake.

**Method:**

A mixed methods research design was employed. Participants (*n* = 493) recruited from primary care centers, participated in a face-to-face survey that assessed their knowledge and attitudes toward CRC screening. It was followed by in-depth interviews with the consenting participants (*n* = 16) to explore factors affecting screening uptake. Quantitative data was analyzed via descriptive statistics with Mann–Whitney U and Kruskal-Wallis ANOVA tests applied to examine knowledge differences across sociodemographic variables. Whereas qualitative data was analyzed via thematic analysis.

**Results:**

Low average knowledge scores (10%) and participation levels (12.3%) for CRC screening were evident among the participantsThe majority of the participants held discouraging attitudes, indicating various barriers toward CRC screening. Identified themes relevant to factors affecting screening uptake included knowledge deficits, the influence of inherent beliefs, and the inadequate role of healthcare providers (HCPs).

**Conclusion:**

In order to encourage CRC screening among individuals, policymakers need to invest in community awareness and education campaigns that target primary care physicians and adults from all educational backgrounds.

## Highlights

- CRC screening knowledge and uptake is suboptimal in the Emirati population.
- Stigma and lack of risk perception have a strong influence on CRC screening uptake.
- HCPs need to proactively participate in raising CRC prevention awareness.

## 1 Introduction

Colorectal cancer (CRC) is a leading cause of cancer-associated mortality and morbidity worldwide, with incidence on the rise (1, 2). According to Global Cancer Observatory (GLOBOCAN) 2022 estimates, CRC ranked third among the most common types of cancer and second among the most common causes of cancer-associated deaths globally, responsible for 1.9 million new cases and 904,000 deaths (2) respectively. By gender, it was the third most common cause of incidence and mortality in both males and females (2). CRC burden in countries varies by socioeconomic status, with highly developed countries having the highest incidence, which could be attributed to unhealthy lifestyle—specifically, unhealthy diet, sedentary lifestyle, and aging population, among other possible reasons. Additionally, the recent rise in High Development Index (HDI) levels, especially in the transitioning countries, has led to lifestyle changes such as inappropriate diet, lack of physical activity, smoking and increased body weight. These changes, along with lack of adequate treatment and screening programs, have contributed to increased CRC incidence and mortality rates in the respective countries (3). It is projected that the new CRC cases will reach 2.3 million by 2040 globally (4, 5).

Advancements in CRC screening and treatment have improved the survival rates (6). Evidence indicates that early detection, as carried out via stool tests such as Fecal Occult blood tests (FOBTs) or Fecal Immunochemical tests (FITs) and endoscopy can effectively reduce mortality rates (7). FOBT and FIT detect blood in the stool, however FOBT may provide false positive results due to lack of specificity secondary to use of certain medication and foods. This is not a limitation in case of FIT, yet FIT has not been extensively studied in randomized control trials for effectiveness in CRC reduction. On the other hand, procedures such as colonoscopy are based on visual detection of abnormal growth or adenomas in the colorectal area. Limitations of these tests include discomfort and procedural complications such as bleeding, bowel perforation etc. (8). While opportunistic screening has its own benefits, population-based CRC screening has proven to be more effective, as evident from a U. S study suggesting reduction of 25.5 and 52.4% in CRC incidence and mortality, respectively (9). Thus, implementation of CRC screening guidelines in developed countries like US, UK and Australia has led to increase in incidence rates and decrease in mortality and morbidity rates (7, 10, 11).

CRC screening uptake, especially the Fecal Occult Blood test (FOBT) still remains low till date (12). A study comparing CRC screening uptake in 12 high-income countries with organized screening program, using FIT or FOBT, reported it to range between 7 and 67.7% (13). Research has identified the importance of informed decision making and health literacy to address the issue, although knowledge about CRC risk factors and its screening methods has not been found to be associated with willingness to screen (14–17). Furthermore, extensive research has been conducted worldwide to explore barriers toward CRC screening behaviors in order to improve cancer screening A systematic review summarizing factors affecting CRC uptake in different countries of the world highlighted the effect of numerous determinants including cultural views, lack of awareness, negative perceptions about screening tests, logistic barriers such as lack of time, transportation and scheduling challenges, language hurdles, and role of HCP recommendations and influence of family and friends (18).

Within the Arab world and the Eastern Mediterranean region, the situation is not quite different. According to a study by Navabi and Darvishi (19), CRC accounted for 5.9% of the incidence and 5.8% of the mortality rates in 2012 in the Eastern-Mediterranean region, based on data extracted from Cancer Global Project (19).

GLOBOCAN and World Health Organization reports recommend need for more efforts to intervene, through proper screening and early detection programs, along with the call for further research (20). However, research on CRC and its screening knowledge, attitudes and practices remains limited, with studies carried out mainly in Saudi Arabia (17, 21–23), and relatively few in other countries, including Jordan (24), Iran (25), Palestine (26), Qatar (27), Lebanon (28) and UAE (29). These studies consistently report low knowledge levels of and poor attitude toward CRC screening along with the cultural and religious barriers.

In the UAE, CRC is the third and second main cause of cancer incidence and mortality in both the sexes, respectively, according to the Ministry of Prevention and Health, MOHAP, 2019 (30). Increase in the number of cases led the health authorities to take action and introduce national screening program for colorectal cancer (31). The program, launched in Abu Dhabi in 2014, offers free screening for all national (Emirati) males and females. The screening program recommends a routine colonoscopy every 10 years for adult males and females above 40 years of age or a Fecal Immunochemical Test (FIT) test once a year (31), following the gold standard for CRC screening (8). The program was revised in 2019, which previously recommended FIT every 2 years (32). Individuals with family history of CRC and patients with polyps are highly encouraged to have regular as per the American Cancer society guidelines (11).

In the UAE, evidence on the topic is also scarce, with few studies focusing on clinicopathological aspects and prognosis of diagnosed CRC cases (33, 34). Another study has assessed knowledge of CRC risk factors and its screening methods among adults attending a hospital setting in Ajman, UAE, using a survey (35). Accordingly, this study aimed to assess awareness, practices, and attitudes toward CRC screening, along with the exploration of factors affecting screening uptake, among the residents of the Abu Dhabi Emirate, in the UAE.

## 2 Materials and methods

### 2.1 Study design

This study followed a convergent parallel mixed methods research design including a cross-sectional survey to quantitatively assess CRC screening knowledge, uptake, and attitudes, as well as in-depth interviews to qualitatively explore barriers influencing CRC screening. The method refers to type of mixed methods study whereby both data collection processes are conducted concurrently (36). The data analysis for each dataset is carried out independently and results are then combined to get a better and complete picture of the phenomenon. The study components were conducted and are being reported in accordance with the STROBE and COREQ guidelines, respectively (37, 38).

### 2.2 Target population and data collection

For the quantitative part, participants were conveniently sampled from waiting areas of primary health care clinics in the city of Al Ain, Abu Dhabi in 2016. Al -Ain occupies the eastern geographical region of Abu Dhabi, the capital and the largest of the seven Emirates of UAE. The total population of Abu Dhabi is approximately 2,900,000 with nearly 80% expatriates. Primary Health care in the region is provided via Ambulatory Healthcare Services (AHS) clinics, operated by Abu Dhabi Health Services Company (SEHA) (39). Of the 38 total AHS clinics present in Abu Dhabi, 20 are present in Al Ain (39). The data was collected from seven AHS clinics, representing different areas of Al-Ain. Participants attending the clinics were approached individually by the members of the research team in the clinic waiting areas and were informed about the study. Those consenting to participate were provided with a hard copy of the questionnaire. Where required, the questionnaire was filled with the support of the researcher. Adults aged 30 years and above, able to speak English or Arabic, from Emirati as well as non-Emirati background and able to provide consent, were eligible. Those suffering from cancer and/or receiving treatment were excluded. Adequate sample for this study was statistically estimated, using sample size estimation formula for prevalence study with absolute precision, *n = z*^2^ *p (1-p)/d*^2^, where *n* is the estimated sample size, *p* is the expected prevalence of people with awareness of colorectal cancer obtained from a similar study (20), and *d* is the desired margin of error. The value of *z* used was 1.96 (at a 5% level of significance); anticipated prevalence of CRC screening awareness as reported by a study in the region was 37.4% (22) and margin of error was 5%. The calculated sample size was 343. The final sample size was rounded up to a total of 500 participants in order to accommodate for anticipated non-completeness.

The questionnaire was developed to assess knowledge, attitudes and practices, barriers to CRC screening and sources of information. It was based on generic version of Cancer Awareness Measure (CAM) (40), included questions used in previous studies (22, 23, 41) and was adapted according to recommended guidelines within the UAE (31). The questionnaire was reviewed by 25 experts from fields of public health, medicine and oncology for relevance and cultural suitability. Content validity was conducted using Lawshe’s Method, ensuring inclusion of essential and relevant questions only (42). The questionnaire was then pilot tested by 10 participants to ensure reliability, with the resultant Cronbach alpha value as 0.7. The questionnaire was developed in English, reviewed by experts including 25 health care and academic professionals and pilot tested among 10 participants in primary care centers. The English version was translated to Arabic, back translated and checked for accuracy by two team members and pilot tested by 10 more participants. It consisted of four sections: The first section addressed questions on sociodemographic (age, marital status, residence, nationality, residence, residence, socioeconomic and educational status) and health related (pre-existing medical conditions, medication and alternative medicine usage history, family history of cancer, regular visit to a doctor, previous invitation by a doctor for screening test) characteristics. The next section assessed their CRC screening knowledge (Colonoscopy and FOBT), practices (uptake and doctor’s recommendation) and attitudes. The last section enquired about the participants’ sources of information on prevention of CRC. The main outcome measures were CRC screening knowledge and CRC screening (FOBT and colonoscopy) uptake. For CRC screening knowledge, 10 questions related to CRC screening methods were used. Correct response to each question was assigned a score of one, with a score of zero for incorrect response. The aggregate sum of the scores was then computed and converted into percentages to get the final percentage knowledge score for each participant. Other outcome measures included participants’ attitudes toward CRC screening and reported barriers. Data collection was completed over 8 months. Data was collected face to face by medical students, trained in data collection.

The qualitative part of the study included face to face, in depth interviews with a subgroup of participants from the survey. Participants were purposefully sampled, with individuals from both the genders, aged 40 years and above, willing to take part in the interviews, eligible to participate. After participants completed the survey, they were requested to participate in the face-to-face interview. Those indicating willingness were interviewed based on time convenient for them. The interviews were carried out face to face, in Arabic, by a research assistant and two medical students trained in conducting qualitative interviews. The semi structured interview guide, focusing on exploration of factors that could affect CRC screening, was developed based on a thorough literature review. It included topics on participants’ general views on cancer, knowledge of colorectal cancer, its screening modalities, screening participation and experiences; and possible factors that could affect screening uptake. The guide included probes to allow more flexibility and freedom of expression from the participants. The guide was reviewed by two experts in the field of health promotion to ensure relevance and comprehensiveness of the questions. It was also subsequently pilot tested with two participants to ensure the structure and soundness of the questions. The sample size for the qualitative component was determined using theory of saturation, a concept indicating the need to stop data collection at a point when no new ideas or information emerge (43). After each interview was conducted, one of the analysts assessed the transcript to determine if new content was present. Overall, 50 participants were approached, of which majority refused to take part due to nature of the topic and hesitation in speaking about it. The interviews lasted between 30–40 min and were audio recorded after obtaining consent from the participants. After a total of 16 interviews, saturation was reached.

### 2.3 Ethical considerations

Ethics approval was obtained from the Institutional Review Board. Written consent was obtained from all participants for survey as well as the interviews, after they had been provided the information about the study. For participants unable to read, the information was read out. The participants were ensured about the confidentiality of data and their right to withdraw if they did not want to continue with the study. The details of participants were de-identified.

### 2.4 Data analysis

For the quantitative data, Statistical Package for Social Sciences (SPSS), version 28 (IBM Corp., Armonk, N. Y., United States) was used for the analysis (44). Categorical variables were summarized and presented as frequencies and percentages. The unpaired t-test and one-way ANOVA test were used, at a 5% level of significance, to compare colorectal cancer screening knowledge scores across dichotomous and polychotomous independent variables, respectively.

For the qualitative interviews, audio recordings were transcribed followed by translation of transcripts to English. The transcripts were also back translated and checked for accuracy by the senior researcher. Additionally, the findings were shared with the participants to verify their accuracy and ensure that they resonate with the experiences of participants. The transcripts were then analyzed using inductive thematic process where data led to extraction of themes rather than devising themes based on pre-existing framework or theory. The process involved reading and re-reading of the excerpts to gain familiarity with the content, followed by identification of meaningful segments of data to form initial codes. Similar codes were categorized to constitute subthemes, which were further refined and grouped together to assign themes. These themes thus represented the overarching patterns within the data (45). Analysis was carried out by two independent members of research team to ensure reliability of study findings and any disagreement was resolved by mutual discussion during team meetings including lead researcher, held regularly. The inclusion of multiple researchers in data analysis and interpretation ensured prevention of any kind of bias, and thus the validity of the qualitative study. The results were presented along with direct quotes from participants to warrant their perspectives were accurately captured and authentically conveyed. The quotes are labeled, based on the number allocated to the participant, in the order they were interviewed.

## 3 Results

### 3.1 Quantitative—cross sectional study

#### 3.1.1 Demographic characteristics of the participants

About four in every five (80.7%) participants were between the ages of 30 and 49 years (Table 1). Majority of the participants were female (67.1%), married (69.2%), Emirati national (72%), based in Abu Dhabi Emirate (94.6%) and employed (59.2%) (Table 1). Moreover, nearly half of the participants (48.3%) had attained either undergraduate or postgraduate level of education.

**Table 1:** Sociodemographic characteristics of adults taking part in the quantitative survey (*N* = 493).

Demographic characteristics	Frequency	Percentage
Age (years)		
30–39	225	45.7
40–49	172	35.0
50–59	66	13.4
60–69	29	5.9
Gender		
Female	331	67.1
Male	162	32.9
Marital status		
Single	87	17.6
Married	341	69.2
Divorced	47	9.5
Widowed	18	3.7
Nationality		
Emirati	355	72.0
Non-Emirati	138	28.0
Current residence		
Al Ain	431	87.5
Abu Dhabi	35	7.1
Dubai	13	2.6
Others	14	2.8
Employment status		
Unemployed	146	29.6
Own business	55	11.2
Employed	292	59.2
Highest education		
Cannot read and write	18	3.7
Elementary	40	8.1
Intermediate	44	8.9
High school	153	31.0
University	205	41.6
Post-graduate	33	6.7

*Some variables may not add to 493 due to missing values.

#### 3.1.2 Knowledge of colorectal cancer screening

The overall average CRC screening knowledge score among the participants was 16.3 percent (95% CI = 14.75–17.95) with 0 percent and 80 percent as the minimum and maximum scores, respectively. Based on the answers to individual questions, “Have you ever heard about colorectal cancer screening?” and “What is a Fecal occult Blood Test (FOBT)?” had the highest (31.2%) and the lowest (5.1%) correct responses, respectively (Table 2).

**Table 2:** Summary of responses to CRC screening knowledge questions among the survey participants (*n* = 493).

Questions	Correctly answered		Total number of respondents
	*n*	%	
Have you ever heard about colorectal cancer screening?	153	31.2	490
Do you know what the recommendations in UAE for CRC screening are?	76	15.6	487
What is colonoscopy?	129	26.6	485
What is a Fecal occult Blood Test (FOBT)?	24	5.1	475
What is the recommended age to start having a colonoscopy?	44	9.1	486
What is the recommended frequency of a colonoscopy?	25	5.2	482
What is the recommended frequency of a FOBT?	77	15.9	484
When should you visit your doctor for a regular examination?	37	7.6	485
If blood was detected in the stool, does this mean that the person definitely has colorectal cancer?	127	26.2	484
If a person has a lump in the colon or polyp does this mean that he/she has definitely colorectal cancer?	112	23.1	484

The knowledge score was found to be disproportionate across different categories of the participants (Table 3). Males had significantly higher CRC screening knowledge compared to females (19% versus 15%, *p* = 0.046). Similarly, the knowledge, on average, was significantly higher among those below 50 years (18% versus 11%, *p* < 0.001), those residing in Abu Dhabi Emirate (17% versus 7%, p < 0.001), those having attained university level of education (19% vs. 14%, *p* = 0.008), those having a family history of cancer (22% vs. 16%, *p* = 0.004), those who had not been invited for cancer screening (22% vs. 15%, p < 0.001), as well as those having television as a source of information (19% vs. 14%, *p* < 0.003). Conversely, nationality (*p* = 0.116), employment status (*p* = 0.613), marital status (*p* = 0.521) and regular medical visits (*p* = 0.241) were not statistically associated with CRC screening knowledge.

**Table 3:** Differences in CRC screening knowledge scores among various sociodemographic categories of survey participants.

Characteristic	Knowledge score (%)		*p* value
	Mean (95% CI)	SD	
Gender			**0.046**
Female	15.26 (13.13–17.18)	18.77	
Male	18.58 (16.02–21.14)	16.49	
Age			**<0.001**
<50	17.63 (15.83–19.44)	18.28	
≥50	10.63 (7.38–13.88)	15.97	
Nationality			0.116
Emirati	15.55 (13.68–17.42)	17.91	
Non-Emirati	18.41 (15.07–21.28)	18.49	
Residence			**<0.001**
Abu Dhabi Emirate	16.89 (15.16–18.48)	18.26	
Others	7.04 (2.28–11.80)	12.03	
Employment status			0.613
Unemployed	16.99 (14.02–19.95)	18.13	
Employed	16.08 (14.07–17.89)	18.11	
Education			**0.008**
Below university	14.27 (12.06–16.49)	17.93	
University	18.57 (16.14–20.74)	18.06	
Marital status			0.521
Single	15.75 (17.80–19.70)	18.53	
Married	17.01 (14.99–18.82)	18.03	
Divorced	14.47 (9.07–19.87)	18.39	
Widow	11.67 (3.27–20.07)	16.89	
Regular medical visit			0.241
No	15.66 (13.61–17.50)	17.86	
Yes	17.68 (14.85–20.50)	18.54	
Cancer family history			**0.004**
No	15.50 (13.73–17.12)	17.84	
Yes	21.82 (17.16–26.48)	18.97	
Screening invitation			**<0.001**
No	22.35 (18.83–25.87)	17.92	
Yes	14.78 (12.92–16.46)	17.84	
TV as a source of information			**0.003**
No	14.29 (12.11–16.21)	17.74	
Yes	19.22 (16.72–21.73)	18.25	

#### 3.1.3 Attitude

**Figure 1:**
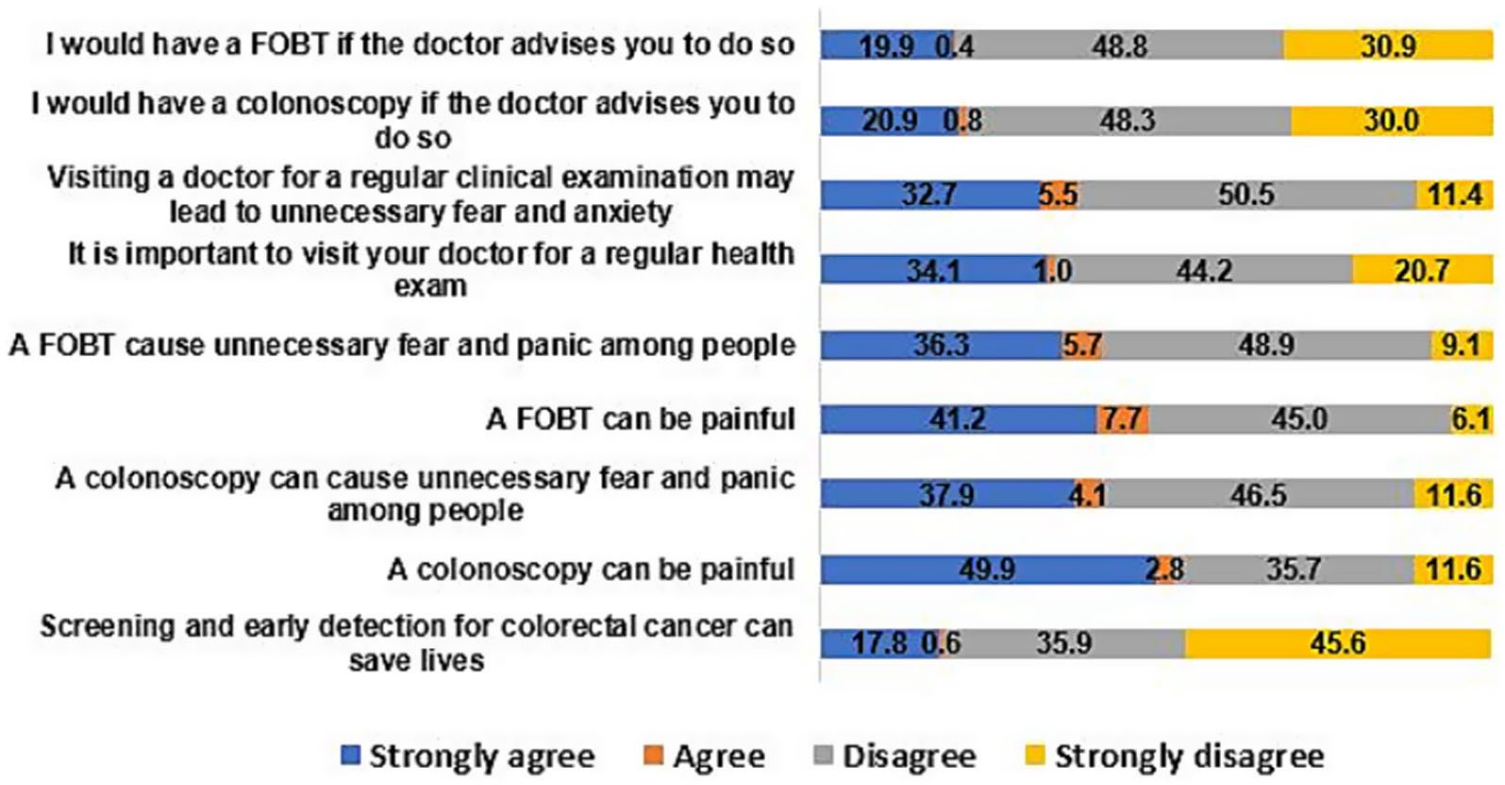
Participants’ attitude toward colorectal cancer screening expressed in %age (*N* = 494).

The participants’ attitudes toward CRC screening is graphically presented in Figure 1. Many of the participants, at least one in every five, believed that colonoscopy (52.7%) and FOBT (48.9%) can be painful, and can cause unnecessary fear and panic (42%). Moreover, about one-fifth of the participants agreed that they would have a colonoscopy (21.7%) or FOBT (20.3%) on doctors’ advice. Only a few of the participants (18.4%) believed that screening and early detection for colorectal cancer can save lives.

#### 3.1.4 Practice of and barriers to colorectal cancer screening test

Only a minority of the participants (12.4%) reported having a colonoscopy in the last 10 years with reference to the survey period. A similar proportion of the participants (12.4%) reported having FOBT and most (12.2%) of them had it conducted in the last year *before the survey*.

**Figure 2:**
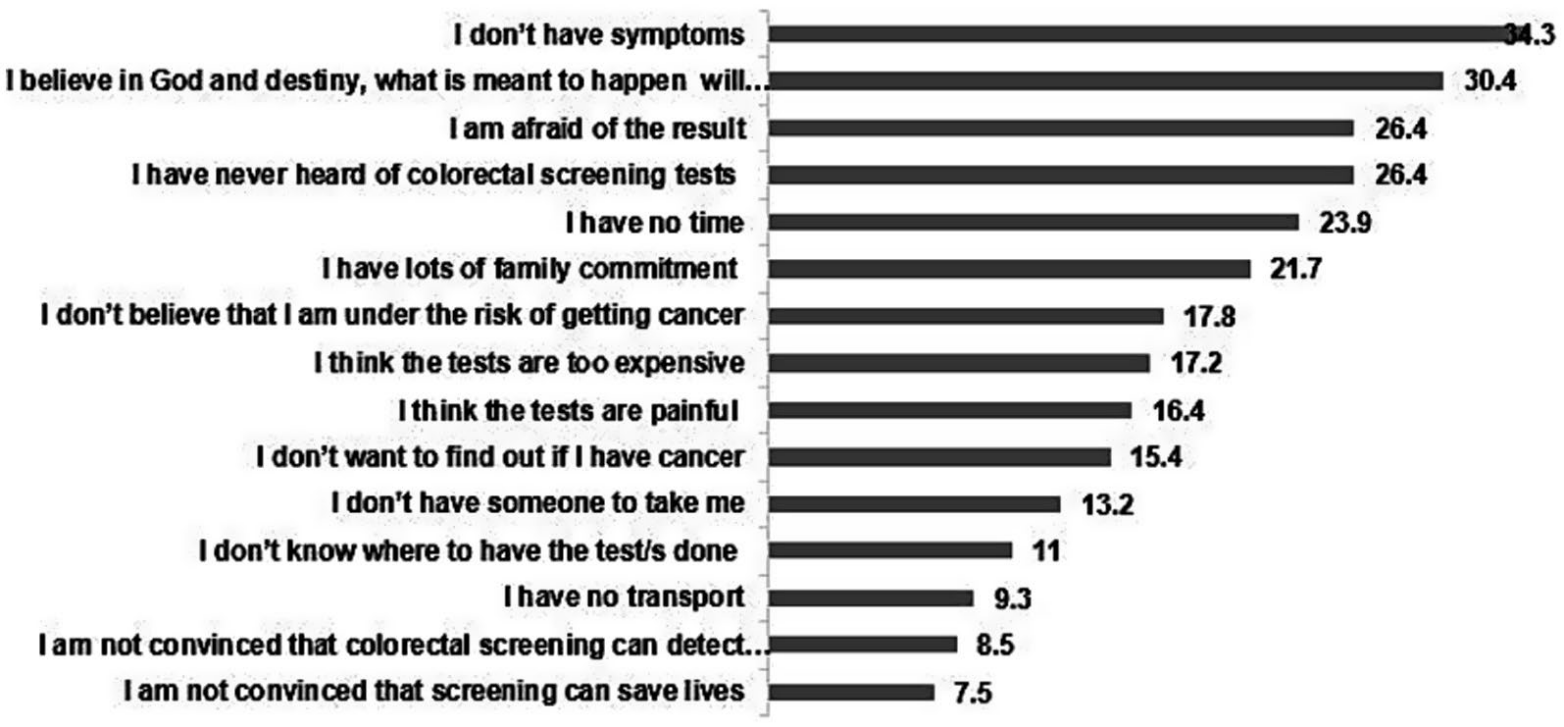
Reasons for lack of participation in the colorectal cancer screening tests expressed in %age (*N* = 493).

The most common reason for not having a CRC screening test was lack of symptoms (34.3%), followed by perceived belief in destiny (30.4%) (Figure 2). Other frequent reasons included being afraid of test results (26.4%), lack of knowledge about the screening tests (26.4%), lack of time (23.9%), family commitments (21.7%). The least common reasons were lack of means to reach the testing center (13.2%), lack of knowledge about the place of testing (11%), lack of transport (9.3%), lack of conviction about the test efficiency in cancer detection (8.5%) and that the screening can save lives (7.5%).

#### 3.1.5 Sources of information

**Figure 3:**
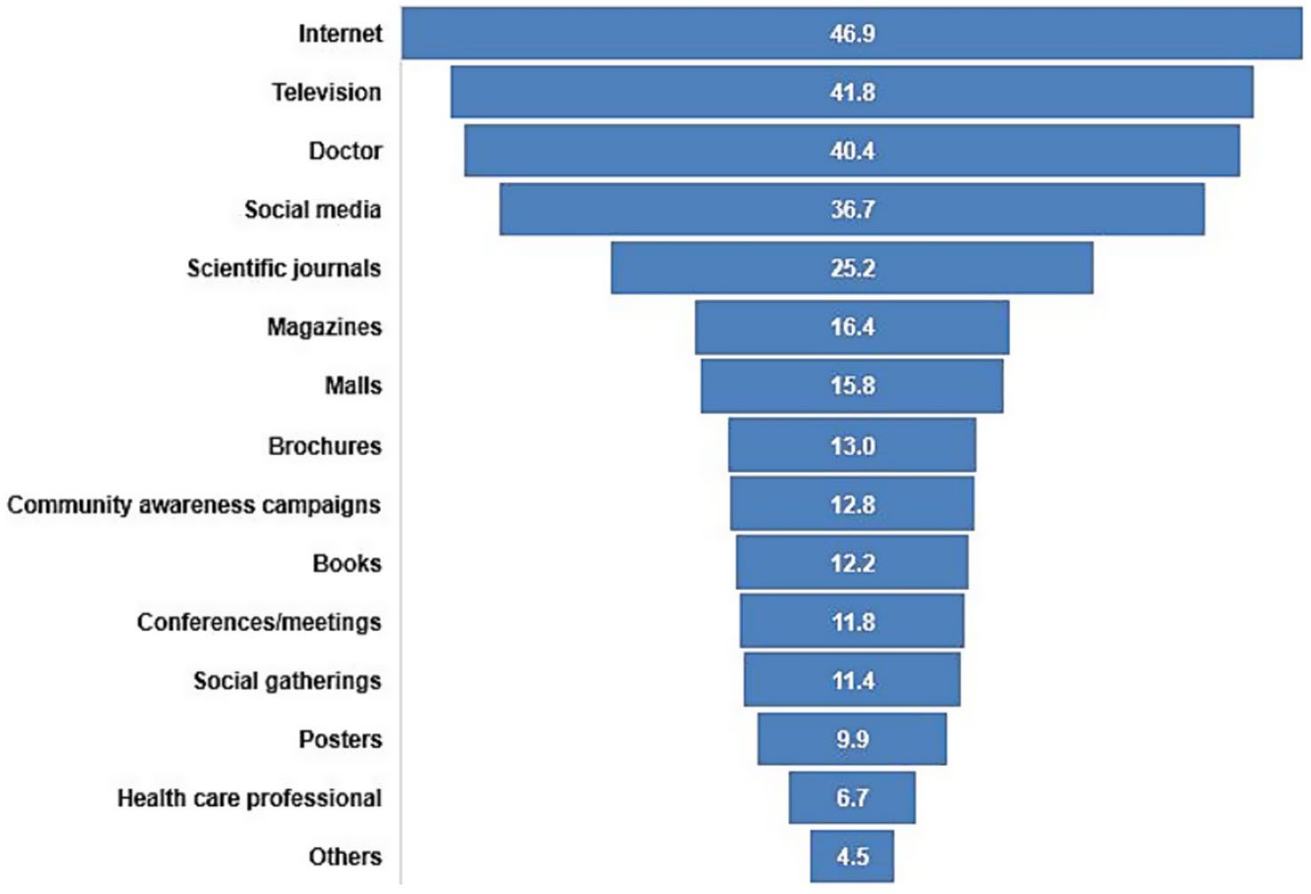
Proportion of participants by sources of information regarding colorectal cancer screening (*N* = 493).

The distribution of the sources of information among the participants is depicted in Figure 3 with the internet (46.9%), television (41.8%), and doctor (40.4%) being the most common sources of information as reported by the participants.

### 3.2 Qualitative—in depth interviews

The age range of the 16 interviewed participants was 40–75 years. The majority (*n* = 12) were females whereas the rest were males. All the interviewed participants were married, and the number of children ranged between zero to 21. Of the interviewed participants, eight had not heard of colonoscopy, with only two having had it done. and six had not heard about the FOBT. Major themes on factors influencing CRC screening that emerged, included lack of awareness, impact of intrinsic concepts and beliefs on screening behavior and inadequate HCP involvement (Table 4). The resulting themes are explained as follows, with representative quotes elaborating the participants’ perspective.

#### 3.2.1 Lack of awareness

One of the major barriers that could be delineated from conversation with participants was insufficient awareness and knowledge of the cancer type as well its screening methods. Although majority of the participants were knowledgeable of the term “cancer” in general, and had also heard the word “colon cancer,” they did not have sufficient information on details of the disease. They were not aware of the actual organ involved, as many participants misconstrued it as “somewhere in the stomach” (P#10) or “something in the intestines” (P#3). The reason for lack of awareness was outlined as being a less commonly brought up cancer, compared to other types of cancer:

“We, in general, women who are housewives, know the most about breast cancer. Because one of my friends got sick, she went to the appointment and found out she had breast cancer. It is the most common type of breast cancer” (P#5).

When asked about the CRC burden, participants remained unaware. However, participants did refer to signs and symptoms that it may lead to, including indigestion, diarrhea, and mass in the intestine.:

“You know one of my neighbors, she had pain in the stomach, and she lost a lot of weight. They took her to the hospital. She was treated for one year but then she died” (P#13).

They were uncertain of the treatment options and when asked about screening methods, it was commonly referred to as testing through blood sample. Few participants also viewed x ray as a way of detecting the disease. Nearly half the patients were not aware of the stool test. Of those that were aware misconceived the purpose, as one female participant explained:

“Yes, they check if there are worms” (P#12).

Likewise, the majority were not aware of colonoscopy, with few participants confusing it with an endoscope used from above (gastroscope), to check gastric lesions.

#### 3.2.2 Role of inherent beliefs

Various innate beliefs reflected in participants’ perspectives regarding screening attendance. Of them, lack of risk perception was predominant. Participants did not consider CRC as a significant threat as they did not have any problem, nor had anyone had in their family. They were of the view that medical attention needs to be sought only when one is experiencing symptoms or is feeling unwell. One of the participants elaborated:

“I only go to the hospital when there is a problem, for a specific reason. Why would I have endoscopy if I don’t have any problem” (P#11).

Thus, for them concept of accessing preventive healthcare was linked to presence of signs and symptoms denoting the ailment. Few participants also referred to use of alternative treatments and herbal remedies:

“I’m a patient person, I try myself with herbs first, then I go to the hospital. I don't go without a reason” (P#6).

Another common attitude exhibited by the participants was having fatalistic beliefs, considering all diseases to be predestined and inevitable. According to one of them:

“God writes it for a human being. God sends down disease or erases it. Whoever God gives a long life will get treatment. And for whoever God does not write life, then this is by the command of God Almighty. You can’t get well if God does not will” (P#7).

Element of shame and embarrassment was also expressed in having the CRC screening, especially colonoscopy. Of the participants that were aware of colonoscopy, most indicated that they would not prefer to get the test from below, owing to the position of private parts:

“I refused to do an endoscopy from below. I don't want it from under honestly. I honestly don't accept it. I'm ashamed to let a man do it for me” (P#9).

Few participants that misinterpreted colonoscopy for gastroscopy were pleased to have the procedure conducted from above, instead of from below, as the former was disgusting according to them.

**Table 4:** Various themes and subthemes as identified during thematic analysis.

Theme	Subtheme
Lack of awareness of colorectal cancer	General awareness of the term “cancer” Uncommon type of cancer Lack of detailed and specific knowledge Recognition of signs and symptoms Understanding of screening modalities
Role of inherent beliefs	Low risk perception Preventive healthcare, an uncommon concept Preference for alternative medicine Fatalistic attitudes Stigma related to procedures
Lack of HCP involvement	Insufficient recommendations by doctors Focus on treatment rather than prevention Alternative channels for information Having trust in doctor’s advice

#### 3.2.3 Lack of HCP involvement

One of the most common factors outlined for nonattendance toward CRC screening uptake among participants, was lack of recommendation by their healthcare practitioners. It seemed obvious that they were not getting enough information from the HCPs. One of the participants when asked if the doctor has recommended him the test, responded:

“Doctor has not asked me to do any colon related tests, he just does BP checks” (P#14).

This was also evident through elaboration of participants when enquired about their source of information. Social media and television were the most common means:

“On TV, I hear people's news, TV stories. Doctors do not tell but children speak” (P#7).

It was apparent that participants consider the doctor as trusted sources of information provision and without their active suggestion they do not consider CRC screening necessary. Another reason that was brought up was the inadvertent behavior of HCPs, causing the participants to seek medical help only when strictly required and discouraging seeking preventive healthcare:

“I feel that doctors don't encourage you to come without a reason. Their reaction is that one should not come without reason” (P#8).

Hence it was evident that participants, without guidance from HCPs, were unaware of the potential benefits of CRC screening as well as unfamiliar with the specific guidelines and recommendations for their age group or risk factors. However, most participants indicated the willingness to attend such programs if recommended by HCPs. They were also appreciative of the available resources provided by their government, available through social media.

### 3.3 Integration of findings from qualitative and quantitative components

The incorporation of results from the questionnaire with those from the interviews enabled data triangulation and validation of study findings. The outcome from the survey, that participants had low levels of CRC screening knowledge and uptake, was corroborated by views brought up in the interviews. The conversations with the participants detailed that awareness of the cancer site and purpose for screening is severely lacking, which leads to insufficient understanding of recommendations as well as lack of motivation for test uptake. Inherent factors such as insufficient perception of disease severity or seeking healthcare when healthy solely for the sake of screening, further provided the context for poor uptake and affecting barriers. Report from the survey that participants who had been recommended by HCPs had more CRC screening knowledge, was also confirmed by interview findings, outlining HCPs’ behavior favoring clinic attendance mostly when suffering from disease.

## 4 Discussion

This research study explored CRC knowledge, uptake and behaviors using a mixed methods study approach. Results suggest low knowledge of CRC screening modalities, as well as poor screening uptake, influenced by diverse attitudes. Reasons for lower knowledge and uptake were further explored to reveal role of misconceptions, inherent behaviors and healthcare provider related issues, acting as barriers toward screening.

CRC screening knowledge in general (31.2%) as well in specific about FOBT (5.1%) and colonoscopy (6%), as indicated in our study, was very low. When compared with other studies within the region, it was in consistency with evidence from similar studies conducted in Saudi Arabia (37.4%) and Jordan (24%) (22, 46). The factors associated with high knowledge including higher education level, and family history of CRC, as indicated in our study, were similar to those reported in another study conducted in Saudi Arabia. However, the latter found females to be more knowledgeable than males as opposed to in our study (47). It is important to understand that lack of knowledge concerning origin of the disease as apparent in current qualitative exploration, hinders individuals from recognizing potential symptoms and seeking timely medical attention, thus leading to premature mortality. It is noteworthy that both the survey and interviews reported lack of comprehension regarding purpose of screening tests and details of screening guidelines. Another study conducted in Ajman, another Emirate in UAE, assessing knowledge on CRC risk factors and screening among adult population indicated it to be 18.3 and 5.9%, respectively (35). This indicates a dire situation on awareness of CRC within the country as ignorance may result in underestimation of the significance of screening in preventing and detecting CRC and lack of compliance. It is notable that mean age for getting diagnosed with CRC in Middle East has been reported to be 10 years less than that in the U. S. and within UAE it has been reported that 22% of the colon cancer occurs in patients less than 40 years of age (48, 49). Thus, educating individuals about the importance of CRC is essential as it can encourage them to prioritize their health and take proactive steps toward prevention and early detection. Healthcare providers should engage in open and clear discussions about the importance of screening, the rationale behind recommendations, and the potential benefits of early detection. Patient education materials and discussions need to emphasize that screening is not solely for diagnosing diseases but also for preventing or detecting conditions at an early and treatable stage.

The screening uptake for colonoscopy (12.4%) and FOBT (12.4%), as revealed by our study is also low. A study conducted in Abu Dhabi, that utilized health care records of adult Emiratis visiting ambulatory healthcare centers in Abu Dhabi, UAE during 2015–16, reported CRC screening uptake be 23.5% for FOBT and 13.5% for follow-up colonoscopy among individuals with positive FOBT results (29). It is comparatively similar to our findings. Moreover, studies reporting uptake in other countries of the region suggest similar poor uptake rates such as 6% in Oman, 15.1% in Lebanon and 15.2% in Saudi Arabia (17, 28, 50).

Interviews as well as the survey highlighted commonly prevalent barriers toward screening uptake among the participants. These, include modifiable factors such as lack of risk perception, fatalistic beliefs, fear of pain and discomfort, fear of test results and absence of preventive health seeking practices, and have been reported in the literature previously, corroborating our findings (18). Additionally, feelings of shame or embarrassment were also brought up in our study as a barrier to attending CRC screening. It is noteworthy that studies conducted to understand preference of CRC screening method in Saudi Arabia reported that participants preferred FICT and FOBT than colonoscopy on account of lack of embarrassment and practicality of the procedure (51, 52). It is essential to understand that shame and stigma has existed as an important barrier to cancer screening when particularly involving intimate or sensitive areas of the body (53). Therefore, emphasis on normalizing the talk around colorectal cancer, easing the shame and stigma that surrounds the willingness and respecting patient choices for methods of preference can be the key to improve screening uptake. It is noteworthy that there are certain non-modifiable factors such as cultural norms, religious beliefs, and cancer-related stigma, as observed in our study, which also influence attitudes toward CRC screening and contribute to avoidance. However, when compared with barriers reported in western countries, high screening cost and lack of insurance coverage are most commonly indicated as reported in the literature (60).

The quantitative results, aided by the qualitative findings highlighted that HCPs’ role in providing adequate information was not being achieved. A study on knowledge of adult Emiratis regarding CRC revealed that CRC screening was not recommended by HCPs to 95% of participants (54). Although other studies on HCPs’ knowledge and attitudes toward CRC screening within the region suggest low knowledge but positive attitudes (55), such research needs to be carried out in UAE to assess primary health physicians’ perspectives on CRC prevention. A study, in 14 Asian pacific countries, assessing the knowledge and cues to actionsfor CRC screening, highlighted physician involvement as a key predictor (41). Other studies have also consistently outlined the critical role of physicians in promoting CRC screening, concluding that enhancing physician awareness is essential (15). Research indicates that improving cancer literacy and practitioner training to provide patients prompts boosts screening rates (56, 57). Literature also suggests that interactions between physicians and patients rather than one way interaction usually led by the physicians can improve the discussion about CRC screening (58). Moreover, it has been confirmed that participation in CRC programs is highly dependent on people’s perceptions about barriers and benefits of such screening (59). In short effective communication between healthcare practitioners and patients is essential and lack of recommendation by healthcare practitioners can be a significant reason for screening nonattendance. To address this barrier, healthcare providers should actively engage in patient education, provide personalized recommendations based on individual risk factors, and foster a collaborative approach toward healthcare decision-making. This can ultimately lead to improved screening rates and better health outcomes for patients.

A potential limitation of the study is the limited generalizability of results to the overall UAE population, primarily attributable to the convenient sampling method employed. Furthermore, there is an inability to establish causal relationship between CRC screening knowledge and associated factors due to application of a cross-sectional study method for the survey. Additionally due to reliance on self-reported data, findings may be subject to recall and social desirability biases Although, the questionnaire was thoroughly assessed for content validity and reliability, the construct validity was not measured. Future research employing a fully validated questionnaire would be beneficial. An important limitation is that we could not go through the full process of validation using factor analysis nevertheless, we have conducted content validity and Cronbach alpha There is also a potential of researcher bias, as interpretation of qualitative data is dependent upon researcher’s views and beliefs. However, it was overcome by consistent research team meetings for theme discussion. Nevertheless, use of qualitative interviews, along with quantitative questionnaires, allowed for better understanding and triangulation of the results, enhancing study reliability. The study reports findings from data collected in 2016. Although the data can be considered old, it needs to be published in order to provide a baseline for future studies that include assessment of CRC screening knowledge after the screening program in UAE was revised in 2019 as well as for intervention implementation.

## 5 Conclusion

This study provided an insight into the knowledge, attitudes and behaviors regarding CRC screening among residents of Al-Ain, UAE. Results imply that low knowledge and uptake rates, under the influence of varied attitudes and behaviors, prompt action. Considering reluctance to CRC screening as a social and behavioral challenge, steps such as raising awareness, promotion of the benefits of early detection and preventive healthcare and dispelling underlying misconceptions are required. This needs to be tackled through channels not keenly involved in the process such as HCPs. Public health campaigns and tailored messaging can also be effective in changing these beliefs and promoting proactive CRC screening, leading to greater participation in screening initiatives. Future recommendations include the need for more studies in different Emirates and as well as the need for policy makers to invest in more awareness campaigns about CRC and screening availability.

## Data Availability

The raw data supporting the conclusions of this article will be made available by the authors, upon reasonable request.
